# Creative leisure activities, mental health and well-being during 5 months of the COVID-19 pandemic: a fixed effects analysis of data from 3725 US adults

**DOI:** 10.1136/jech-2022-219653

**Published:** 2023-02-27

**Authors:** Jessica K Bone, Daisy Fancourt, Jill K Sonke, Meg E Fluharty, Randy Cohen, Jenny B Lee, Anthony J Kolenic, Heidi Radunovich, Feifei Bu

**Affiliations:** 1 Research Department of Behavioural Science and Health, Institute of Epidemiology & Health Care, University College London, London, UK; 2 Center for Arts in Medicine, University of Florida, Gainesville, Florida, USA; 3 Americans for the Arts, Washington DC, Washington, USA; 4 Family, Youth and Community Sciences, University of Florida, Gainesville, Florida, USA

**Keywords:** COVID-19, DEPRESSION, EPIDEMIOLOGY, LONGITUDINAL STUDIES, MENTAL HEALTH

## Abstract

**Introduction:**

We investigated whether changes in engagement in home-based creative activities were associated with changes in depressive symptoms, anxiety symptoms and life satisfaction during the COVID-19 pandemic, aiming to replicate findings from the UK in a USA sample.

**Methods:**

3725 adults were included from the COVID-19 Social Study in the USA, a panel study collecting data weekly during the COVID-19 pandemic. We measured engagement in eight types of creative leisure activities on the previous weekday between April and September 2020. Data were analysed using fixed effects regression models.

**Results:**

Increased time spent gardening was associated with reductions in depressive and anxiety symptoms and enhanced life satisfaction. Spending more time doing woodwork/DIY and arts/crafts were also associated with enhanced life satisfaction. However, more time watching television, films or other similar media (not for information on COVID-19) was associated with increased depressive symptoms. Other creative activities were not associated with mental health or well-being.

**Conclusion:**

Some findings differ from evidence obtained in the UK, demonstrating the importance of replicating research across countries. Our findings should also be considered when formulating guidelines for future stay-at-home directives, enabling individuals to stay well despite the closure of public resources.

**WHAT IS ALREADY KNOWN ON THIS TOPIC**

- During the COVID-19 pandemic in the UK, spending more time doing outdoor activities and creative hobbies was associated with improvements in depression, anxiety and life satisfaction.

**WHAT THIS STUDY ADDS**

- In the US, increases in time spent doing gardening, woodwork and DIY, and arts and crafts were longitudinally associated with improvements in mental health and wellbeing during the COVID-19 pandemic.

**HOW THIS STUDY MIGHT AFFECT RESEARCH, PRACTICE AND/OR POLICY**

- Our findings demonstrate the importance of replicating research across countries, exploring how cultural or contextual nuances affect the associations between creative engagement and health outcomes. Future guidelines for stay-at-home directives should consider the role of creative leisure activities.

## Introduction

Given the widespread stay-at-home orders during the COVID-19 pandemic, identifying activities that people engaged in at home and that helped to support their mental health and well-being is a key priority.^1^ In the UK, spending more time doing outdoor activities (eg, gardening, exercising), as well as creative hobbies (eg, painting, digital arts), was associated with improvements in depression, anxiety and life satisfaction.^2^ However, it is unclear whether these findings from the UK will generalise to the USA. The USA differs from the UK in terms of social, cultural and demographic context, the inconsistent COVID-19 restrictions across states in the country, and varying job subsidy schemes for cultural workers during the pandemic.

In the USA, cross-sectional studies have found that self-reported decreases in leisure activities, compared with before the pandemic, were associated with higher depressive symptoms and lower well-being.^3^ Some older adults also reported doing hobbies and other creative activities, such as cooking and baking, listening to music and reading to help them cope during the pandemic.^4^ Elsewhere, also in cross-sectional surveys, people have reported increases in creativity during the pandemic,^5–8^ with increased time spent on creative activities such as crafts, fine arts, music and performing arts all associated with enhanced well-being.^6^ In correlation analyses of another cross-sectional survey of Australian adults, more time spent on artistic creative activities was correlated with lower anxiety and depressive symptoms and less loneliness.^9^ However, the longitudinal associations between creative leisure activities and mental health and well-being during the pandemic outside of the UK remain unclear.

Therefore, in this study, we aimed to replicate findings from the UK in a USA sample, with a focus on creative activities. There is extensive prepandemic longitudinal evidence that creative activities are beneficial for mental and physical health.^10^ As opportunities for engaging in creative activities changed dramatically with the onset of the pandemic,^11 12^ this may have limited their benefits. We, therefore, examined whether changes in engagement in home-based creative leisure activities were longitudinally associated with mental health and well-being during the COVID-19 pandemic in the USA.

## Methods

### Sample

This panel study was a USA extension of the UK COVID-19 Social Study run by UCL (https://github.com/UCL-BSH/CSSUserGuide; online supplemental file 1). Adults in the USA completed an online survey weekly between April and September 2020 for a maximum of 12 waves. A heterogeneous sample was recruited using a snowballing approach with a focus on reaching diverse populations. National social, health and arts organisations and networks shared the study invitation through email lists and social media. We included a total of 3725 participants (22 190 observations; observations per person: mean=6, median=5, ranging from 2 to 12). More information on sample selection is available in online supplemental file 1.

10.1136/jech-2022-219653.supp1 Supplementary data

### Measures

#### Leisure activities

Participants reported how long they had spent on the last weekday engaging in (1) reading for pleasure, (2) arts/crafts activities (eg, painting, creative writing, sewing, playing music), (3) digital arts activities (eg, streaming a concert, virtual tour of a museum), (4) gardening, (5) watching television, films, Netflix or similar, (6) listening to the radio or music, (7) doing DIY (do-it-yourself), woodwork, metal work, model making or similar and (8) another hobby not already mentioned. Time spent on each activity was categorised as none, low (<30 min) or high (≥30 min).

#### Mental health and well-being

Depressive symptoms were measured using the Patient Health Questionnaire (PHQ-9).^13^ Across waves, Cronbach’s α for the PHQ-9 ranged from 0.87 to 0.89. Anxiety symptoms were measured using the Generalised Anxiety Disorder Assessment (Cronbach’s α ranged from 0.91 to 0.92).^14^ Life satisfaction (evaluative well-being) was measured with one question ‘Overall, in the past week, how satisfied have you been with your life?’.

#### Covariates

Most demographic, socioeconomic and health-related covariates were measured at baseline in this study (age, gender, race/ethnicity, education, marital status, employment status, household income, urbanicity of home area, keyworker status (whether participants were a critical employee who provided an essential service throughout the pandemic), diagnosed mental health problem, diagnosed physical health problem or disability). However, several COVID-19-related factors were reported in each wave. We measured (yes, no) whether participants: had ever been diagnosed with or suspected having COVID-19; had been in contact with someone with COVID-19 in the last week; had lost their job, partner lost their job or had a major cut to household income due to changes in employment; were unable to pay bills, rent or mortgage, or were evicted or lost accommodation; had face-to-face contact or a phone or video call with another person for 15 min or more. We also measured whether participants’ isolation status (living as normal or almost normal, only going out for essentials or exercise, fully isolating).

### Statistical analysis

Fixed effects models tested the longitudinal associations of engagement in creative leisure activities with mental health and well-being. This approach uses only within-individual variation to examine how change in activity engagement is related to change in mental health within individuals over time. All time-invariant factors are accounted for automatically, even if unobserved. We standardised all outcomes and tested three models, using depressive symptoms, anxiety symptoms and life satisfaction as separate outcomes, including all leisure activities simultaneously. We then adjusted these fixed effects models for time-varying covariates.

In sensitivity analyses, we first tested whether the associations between leisure activities, mental health and well-being differed according to baseline employment status. We then included a combined index of time spent on creative hobbies (arts/crafts, digital arts, woodwork/DIY) to allow comparison to previous research.^2^ For all analyses, we weighted data to match the characteristics of the non-institutionalised USA population aged 18 and over. We weighted the final analytical sample according to age, gender, race/ethnicity and education, obtained from the US Census Bureau.^15^

## Results

After weighting, 49% of the sample were aged 30–59 years, 59% were female and 81% were white (table 1). Participants who were older, had fewer depressive and anxiety symptoms, and had higher life satisfaction were more likely to complete more survey waves, although frequency of leisure activity engagement did not differ by participation rates (online supplemental table S1). The most common activity was watching television and the least common was woodwork/DIY (online supplemental table S2). Across the study period, there were substantial changes in activity engagement and mental health and well-being (online supplemental tables S2 and S3).

**Table 1:** Characteristics of the sample at baseline

	Unweighted	Weighted
	Proportion of sample	
Age (years)		
18–29	14%	18%
30–59	53%	49%
60+	33%	33%
Gender		
Male	16%	41%
Female	84%	59%
Race/ethnicity		
White	87%	81%
Black/African American	3%	8%
Asian/Asian American	2%	5%
Mixed Race	3%	3%
Other	5%	3%
Education		
High school or less	3%	14%
Some college	17%	35%
Undergraduate	32%	31%
Postgraduate/professional	49%	20%
Marital status		
Single, never married	20%	26%
Single, divorced or widowed	16%	16%
In a relationship/married, living apart	6%	6%
In a relationship/married, cohabiting	58%	52%
Employment status		
Unemployed	34%	46%
Employed	66%	54%
Household income		
<US$75 000	45%	54%
≥US$75 000	55%	46%
Urbanicity		
City	52%	51%
Town	37%	35%
Rural	11%	14%
Keyworker	23%	22%
Mental health problem	37%	38%
Physical health problem or disability	37%	40%
Had COVID-19 (diagnosed or suspected)	8%	8%
	Mean (SD)	
Depressive symptoms (PHQ-9)	7.60 (5.79)	8.00 (6.27)
Anxiety symptoms (GAD-7)	5.96 (5.22)	5.93 (5.42)
Life satisfaction	5.71 (2.30)	5.52 (2.49)

Note. Mental health problem includes clinically diagnosed anxiety, clinically diagnosed depression, or other clinically diagnosed mental health problem. Physical health problem or disability includes diabetes, heart disease, lung disease, cancer, another clinically diagnosed chronic physical health condition, a disability that affects ability to leave the house, or another disability.

GAD-7, Generalised Anxiety Disorder-7; PHQ-9, Patient Health Questionnaire-9.

### Depressive symptoms

The largest reduction in depressive symptoms was for participants who increased their time spent gardening from none to less than 30 min per day (table 2). More than 30 min per day gardening was associated with a smaller reduction in depressive symptoms. In contrast, increasing time spent watching television from none to more than 30 min per day was associated with increases in depressive symptoms. Adjusting for time-varying covariates did not alter these associations (online supplemental table S4).

**Table 2:** Fixed effects models testing associations between changes in time spent on leisure activities and changes in mental health and well-being across the follow-up period (6 April 2020–6 September 2020)

	Depressive symptoms		Anxiety symptoms		Life satisfaction	
	Coef (95% CI)	P value	Coef (95% CI)	P value	Coef (95% CI)	P value
Reading for pleasure						
Low	0.01 (−0.03 to 0.05)	0.548	0.02 (−0.02 to 0.06)	0.283	0.01 (−0.03 to 0.06)	0.531
High	0.02 (−0.02 to 0.06)	0.311	0.01 (−0.03 to 0.05)	0.610	0.01 (−0.04 to 0.06)	0.648
Arts/crafts						
Low	0.04 (0.00 to 0.08)	0.083	0.01 (−0.02 to 0.05)	0.416	−0.01 (−0.06 to 0.04)	0.730
High	0.01 (−0.03 to 0.06)	0.579	−0.01 (−0.06 to 0.03)	0.621	**0.06 (0.00 to 0.11**)	**0.034**
Digital arts						
Low	0.01 (−0.03 to 0.06)	0.582	−0.01 (−0.04 to 0.03)	0.753	−0.04 (−0.09 to 0.02)	0.180
High	0.04 (−0.03 to 0.10)	0.251	0.04 (−0.01 to 0.09)	0.161	−0.05 (−0.12 to 0.02)	0.166
Gardening						
Low	−**0.07 (−0.11 to 0.02**)	**0.003**	−**0.05 (−0.09 to 0.01**)	**0.015**	0.04 (−0.01 to 0.09)	0.107
High	−**0.05 (−0.09 to 0.00**)	**0.046**	0.00 (−0.06 to 0.05)	0.882	**0.06 (0.00 to 0.12**)	**0.043**
Watching television						
Low	0.04 (−0.01 to 0.09)	0.148	−0.02 (−0.07 to 0.03)	0.457	0.00 (−0.07 to 0.06)	0.948
High	**0.06 (0.01 to 0.10**)	**0.010**	−0.02 (−0.06 to 0.03)	0.479	−0.01 (−0.06 to 0.04)	0.785
Listening to music						
Low	−0.01 (−0.04 to 0.03)	0.725	−0.01 (−0.04 to 0.02)	0.407	0.00 (−0.04 to 0.04)	0.927
High	−0.02 (−0.06 to 0.02)	0.346	−0.01 (−0.05 to 0.02)	0.457	0.00 (−0.04 to 0.04)	0.870
Woodwork/DIY						
Low	0.02 (−0.05 to 0.08)	0.604	0.02 (−0.05 to 0.08)	0.619	−0.04 (−0.10 to 0.03)	0.260
High	0.00 (−0.05 to 0.05)	0.984	−0.02 (−0.07 to 0.03)	0.428	**0.11 (0.03 to 0.18**)	**0.004**
Other hobbies						
Low	0.02 (−0.03 to 0.07)	0.376	0.03 (−0.02 to 0.07)	0.235	−0.04 (−0.09 to 0.02)	0.158
High	0.01 (−0.03 to 0.06)	0.577	0.00 (−0.04 to 0.05)	0.858	−0.02 (−0.09 to 0.04)	0.438

Note. Time spent doing activities measured on the last weekday. Low=less than 30 min doing activity during the day. High=30 min or more spent on activity during the day. Both low and high were compared with doing none of this activity. Outcomes were standardised, so coefficients represent changes in SD units. Bold text indicates p<0.05. Fixed effects analysis does not test the direction of associations, so we cannot conclude that changes in leisure activities lead to subsequent changes in mental health and well-being.

### Anxiety symptoms

Gardening was the only activity associated with changes in anxiety symptoms. Only increasing time spent gardening to less than 30 min per day was associated with lower anxiety symptoms, and this association was maintained after adjusting for time-varying covariates.

### Life satisfaction

Increases in time spent doing arts/crafts, gardening and doing woodwork/DIY were associated with higher life satisfaction. The largest improvement was for participants who increased their time spent doing woodwork/DIY from none to more than 30 min per day. Increasing from no gardening and not doing arts/crafts to more than 30 min per day was also associated with improved life satisfaction. Adjusting for time-varying covariates did not alter these associations.

### Sensitivity analysis

There was no evidence that any of the associations between time on spent on creative leisure activities, depressive or anxiety symptoms, or life satisfaction differed according to employment status (online supplemental table S5). Additionally, total time spent on creative hobbies was not associated with depressive or anxiety symptoms or life satisfaction (online supplemental table S6).

## Discussion

In the USA, during the COVID-19 pandemic, increased time spent on gardening was associated with reductions in depressive and anxiety symptoms and enhanced life satisfaction. Spending more time doing woodwork/DIY and arts/crafts were also associated with improvements in life satisfaction. However, spending more than half an hour per day watching television or other similar media was associated with increases in depressive symptoms.

During the UK COVID-19 lockdown, increases in watching television were also associated with higher depressive symptoms, but not anxiety symptoms or life satisfaction.^2^ Alongside our findings, this provides strong evidence for an association of watching television specifically with depressive symptoms. It is possible that having more depressive symptoms caused people to increase television watching, and not vice versa. In lagged analyses that attempted to remove reverse causality, watching television was not associated with subsequent depressive symptoms during the pandemic in the UK.^2^ However, prepandemic evidence indicates that the association is bidirectional.^16 17^ Future studies should explore the direction of this association and determine whether other coping strategies may be more beneficial. For example, in our study, digital arts were also screen-based sedentary activities, but were not associated with depressive symptoms. These activities could include more health-promoting active ingredients that counteract the negative effects of sedentary screen time.

Evidence that gardening was associated with mental health and well-being is in line with findings from the UK lockdown^2^ and prepandemic research.^18^ During stay-at-home orders, gardening may have provided distance from difficult situations in the home, a change of scenery, access to nature and the feeling of being connected to something bigger.^19^ Gardening includes gentle physical activity, which has extensive benefits for mental health and well-being,^20^ through numerous distinct psychological, physiological and behavioural mechanisms,^21^ particularly during the pandemic.^22^ It was surprising that spending less (vs more) than 30 min gardening was more strongly associated with depressive and anxiety symptoms. People who spent more time gardening may have been doing different types of activities, such as larger projects, which were more stressful. This warrants further investigation.

The lack of evidence for associations between other creative leisure activities and mental health and well-being was unexpected. During the UK lockdown, creative hobbies, reading for pleasure and listening to music were associated with reduced depressive and anxiety symptoms and enhanced life satisfaction.^2^ Creative hobbies included arts/crafts, digital arts and woodwork/DIY. We assessed these activities separately in our main analyses and combined them in a sensitivity analysis. It is unclear why we did not replicate the associations found in the UK, particularly as listening to music and other creative hobbies were effective methods of emotion regulation, which is linked to mental health and well-being, during the pandemic in Australia.^9^ It could be a result of greater heterogeneity in experiences in the USA due to variation in lockdown rules across states, compared with the homogeneity of the UK’s approach early in the pandemic. Alternatively, it could be because of cultural differences in the profile of people using these activities.^23 24^

This study has several strengths, including the large sample, with follow-ups over 12 waves during the first 5 months of the COVID-19 pandemic. We measured longitudinal changes in time spent on leisure activities instead of relying on self-reported changes in engagement (compared with before the pandemic) as in previous cross-sectional studies.^3–9^ Fixed effects models account for observed and unobserved individual heterogeneity. However, they do not address the directionality of associations, so our analyses do not demonstrate whether changes in leisure activities cause changes in mental health and well-being or vice versa. Although we adjusted for COVID-19-related factors, unmeasured time-varying confounders could have influenced our findings. The study did not include a random sample and is thus not representative of the USA population. Data collection began during the pandemic (April 2020) rather than before the pandemic, which may have influenced sample selection. It is unclear how this may have impacted our findings, so analyses should be replicated in a larger more representative sample. As the number of waves completed by participants differed according to their age, mental health and well-being, this may also have caused selection bias. Before weighting, females, people of white race/ethnicity, those with higher education and household income, and those who were employed were over-represented in our sample. Although we weighted data to make it representative of the USA population, we cannot rule out potential biases due to omitting factors associated with survey participation in the weighting process. To match our data to available population statistics,^15^ we excluded participants who did not identify as male or female, but we recognise that gender is not a binary construct.

During the COVID-19 pandemic in the USA, doing gardening, woodwork/DIY and arts/crafts had the most potential to improve mental health and well-being. Despite the similarity of our approach to previous research, our findings differ from evidence obtained in the UK.^2^ This demonstrates the importance of replicating research across countries, as cultural or contextual nuances may affect the associations between creative engagement and health outcomes. Future research should explore the factors underlying these cross-country differences. Our findings should also be considered when formulating guidelines for future stay-at-home directives, enabling individuals to prepare for periods of isolation and stay well despite the closure of many public resources.
