# Supplementary material for: Creative leisure activities, mental health and well-being during 5 months of the COVID-19 pandemic: a fixed effects analysis of data from 3725 US adults
Source: J Epidemiol Community Health. 2023 Feb 27;77(5):293–7. doi: 10.1136/jech-2022-219653 (PMC10086468; doi:10.1136/jech-2022-219653)
Supplement: Supplementary data [file jech-2022-219653supp001.pdf]

Supplementary Materials

Supplementary methods

Sample

The study was launched as a US extension of the UK COVID-19 Social Study run by University College London; a longitudinal study that focuses on the psychological and social experiences of adults living during the COVID-19 pandemic (https://github.com/UCL-BSH/CSSUserGuide). Participants completed an online survey weekly at the start of the pandemic. The US version of the study used the same measures used in the UK COVID-19 Social Study and was delivered in partnership with the University of Florida Center for Arts in Medicine and Americans for the Arts, a non-profit organization whose primary focus is advancing the arts in the US (https://www.americansforthearts.org). In this study, we only included participants from the US extension of the COVID-19 Social Study. Data collection in the US began on 6th April 2020, with participants initially completing a baseline survey, followed by weekly data collection for a maximum of 12 waves.

The COVID-19 Social Study did not recruit a random sample and is thus not representative of the US population. However, it does contain a heterogeneous sample that was recruited using a snowballing approach with a focus on reaching diverse populations. National social, health, and arts organizations and networks shared the study invitation through their email lists and social media. The study was approved by the UCL Research Ethics Committee (12467/005) and the University of Florida Institutional Review Board (IRB202000785). All participants gave informed consent. A full protocol for the primary study is available online at https://github.com/UCL-BSH/CSSUserGuide.

A total of 6,781 participants were recruited across the US, who completed an average of 4.2 waves each (range 1-12 waves; 28,495 observations total). In the present analysis, we focused on participants who had at least two repeated measures between 6th April and 6th September 2020, resulting in a sample of 4,153 participants. We then further restricted the sample to participants who were: over 18; male or female (so that weights could be created); and had complete data on leisure activities and mental health and wellbeing. Although participants reported their gender as male, female, or other, we weighted analyses according to the statistics available from the US Census Bureau (see statistical analyses) who still use a binary measure of gender (male, female; US Census Bureau, 2021), leading to the exclusion of participants reporting their gender as other. This resulted in a final analytical sample of 3,725 participants (total observations: 22,190; observations per person: mean 6.0, range 2-12).

Measures

Leisure activities

Engagement in 35 different activities was measured repeatedly between 6th April and 6th September 2020 using a time diary approach. Participants were asked to focus on a single day and consider how much time they spent on each activity. Given concerns about the cognitive burden of focusing on a ‘typical’ day (which involves aggregating information from multiple days and averaging), participants were asked to focus just on the last weekday. Weekdays were chosen to remove variation in responses due to whether participants completed the survey on weekends. We selected eight creative leisure activities for inclusion in this study.

Participants were asked how long they had spent on the last weekday engaging in 1) reading for pleasure, 2) a home-based arts or crafts activity (e.g., painting, creative writing, sewing, playing music, etc.), 3) digital arts activities (e.g., streaming a concert, virtual tour of a museum, etc.), 4) gardening, 5) watching TV, films, Netflix, or similar (not for information on COVID-19), 6) listening to the radio or music (not for information on COVID-19), 7) doing DIY, woodwork, metal work, model making, or similar, and 8) another hobby not already mentioned. Responses were recorded on a five-point frequency scale, from "did not do" to "did for 6 or more hours". Given the low frequency of engagement in most activities, and consistent with previous research (Bu et al., 2021), we collapsed the time spent on each activity into three categories, none, low (<30 min) or high (≥30 min).

Mental health and wellbeing

Depressive symptoms were measured using the Patient Health Questionnaire (PHQ-9), a nine-item measure with scores ranging between 0 and 27 (Kroenke et al., 2001). Higher scores indicate more depressive symptoms. The standard PHQ-9 was modified in this study to ask about symptoms 'over the last week', instead of 'over the last two weeks', as data were collected weekly.

Anxiety symptoms were measured using the Generalized Anxiety Disorder Assessment (GAD-7), a seven-item measure with scores ranging between 0 and 21 (Spitzer et al., 2006). Higher scores indicate more anxiety symptoms. As with the PHQ-9, questions asked about symptoms 'over the last week' instead of the last two weeks.

Life satisfaction (evaluative wellbeing) was measured using a single question 'Overall, in the past week, how satisfied have you been with your life?', on a scale of 0 to 10. Higher scores indicate more life satisfaction.

Statistical analysis

We used fixed effects models to test the longitudinal associations of engagement in creative leisure activities with mental health and wellbeing. This approach uses only within-individual variation to examine how the change in leisure activity engagement is related to the change in mental health within individuals over time. As individuals are compared with themselves over time, all time-invariant factors (such as gender, age, income, education, and area of living) are accounted for automatically, even if unobserved. Fixed effects models thus control for individual heterogeneity, eliminating potential biases in the estimates of time-variant variables (Allison, 2009). We tested three fixed effects models, using depressive symptoms, anxiety symptoms, and life satisfaction as separate outcomes. All eight types of leisure activities were included in each model simultaneously.

To balance the sample in relation to the target population demographics, we weighted data to match the characteristics of the non-institutionalised US population aged 18 and over. We weighted the final analytical sample according to age, gender, race/ethnicity, and education, obtained from the US Census Bureau (US Census Bureau, 2021), using the Stata user-written package ebalance (Hainmueller & Xu, 2013). To remove extreme variation, weights were trimmed to a maximum of the median plus five times the interquartile range, and then adjusted so that the total summed to the number of participants (Chowdhury et al., 2007; Potter & Zheng, 2015). For comparison, unweighted and weighted demographic characteristics of the sample are presented in Table 1. All analyses were performed using Stata 16 (StataCorp, 2019).

Supplementary tables and figures

Table S1. Unweighted baseline characteristics of the sample according to number of observations per participant.

		Number of waves completed				
		**2**	**3-5**	**6-8**	**9-11**	**12**
Total number of participants		1154	867	633	486	585
Age (years)	18-29	18%	17%	12%	11%	10%
	30-59	52%	59%	48%	42%	53%
	60+	30%	24%	40%	47%	37%
Gender	Male	16%	15%	15%	19%	18%
	Female	84%	85%	85%	81%	82%
Race/ethnicity	White	88%	84%	90%	87%	88%
	Black/African American	3%	4%	1%	4%	2%
	Asian/Asian American	1%	3%	2%	1%	3%
	Mixed Race	3%	4%	3%	3%	2%
	Other	5%	5%	4%	5%	5%
Education	High school or less	3%	2%	2%	3%	2%
	Some college	20%	18%	13%	17%	14%
	Undergrad	33%	32%	30%	33%	34%
	Postgrad/professional	44%	48%	55%	47%	50%
Employment status	Unemployed	37%	31%	34%	43%	38%
	Employed	63%	69%	66%	57%	62%
Household income	<$75,000	47%	44%	45%	46%	42%
	$75,000+	53%	56%	55%	54%	58%
Reading for pleasure	Low	22%	25%	25%	20%	21%
	High	46%	38%	50%	51%	48%
Arts/crafts	Low	17%	15%	26%	13%	12%
	High	26%	28%	32%	26%	27%
Digital arts	Low	10%	11%	14%	8%	6%
	High	9%	12%	13%	9%	8%
Gardening	Low	16%	19%	17%	16%	17%
	High	18%	15%	18%	23%	19%
Watching TV	Low	6%	6%	4%	6%	7%
	High	84%	85%	87%	82%	80%
Listening to music	Low	21%	23%	21%	22%	20%
	High	49%	48%	50%	48%	50%
Woodwork/DIY	Low	6%	5%	6%	4%	4%
	High	7%	7%	6%	6%	5%
Other hobbies	Low	11%	10%	9%	10%	9%
	High	12%	11%	13%	12%	10%
		Mean (standard deviation)				
Outcomes	Depressive symptoms	8.08 (6.04)	8.31 (5.84)	7.57 (5.49)	6.77 (5.59)	6.35 (5.40)
	Anxiety symptoms	6.16 (5.37)	6.67 (5.27)	5.98 (5.09)	5.26 (4.95)	5.07 (5.00)
	Life satisfaction	5.52 (2.33)	5.56 (2.33)	5.78 (2.15)	5.91 (2.39)	6.09 (2.22)

Bone JK, *et al. J Epidemiol Community Health* 2023; 77:293–297. doi: 10.1136/jech-2022-219653

Table S2. Average levels of engagement in leisure activities at baseline and proportion of participants who changed activity status over time.

	Baseline engagement	Proportion who changed activity status
Reading for pleasure		43%
None	33%	
Low	23%	
High	44%	
Arts/crafts		38%
None	59%	
Low	14%	
High	27%	
Digital arts		27%
None	81%	
Low	9%	
High	10%	
Gardening		36%
None	68%	
Low	14%	
High	18%	
Watching TV		34%
None	9%	
Low	6%	
High	85%	
Listening to music		46%
None	27%	
Low	20%	
High	53%	
Woodwork/DIY		20%
None	85%	
Low	6%	
High	9%	
Other hobbies		31%
None	75%	
Low	11%	
High	14%	

Note. Time spent doing activities measured on last weekday. Low = less than 30 mins doing activity during the day. High = 30 mins or more spent on activity during the day.

Table S3. Summary statistics for depressive and anxiety symptoms and evaluative wellbeing

	**Depressive symptoms (PHQ-9)**		**Anxiety symptoms (GAD-7)**		**Life satisfaction**	
**Variation**	Mean	SD	Mean	SD	Mean	SD
Overall	6.61	5.93	5.19	5.29	6.02	2.42
Between individual		5.32		4.85		2.03
Within individual		2.31		2.17		1.17

Note. PHQ-9 score could range from 0-27, with higher scores indicating more depressive symptoms. GAD-7 score could range from 0-21, with higher scores indicating more depressive symptoms. Life satisfaction (evaluative wellbeing) could range from 0-10, with higher scores indicating more life satisfaction.

Table S4. Fixed effects models adjusted for time-varying covariates (COVID-19 status, COVID-19 contact, employment change, financial difficulties, social contact, and isolation status).

	**Depressive symptoms**		**Anxiety symptoms**		**Life satisfaction**	
	Coef (95% CI)	p	Coef (95% CI)	p	Coef (95% CI)	p
**Reading for pleasure**						
Low	0.01 (-0.03, 0.04)	0.704	0.02 (-0.02, 0.05)	0.357	0.02 (-0.03, 0.06)	0.459
High	0.01 (-0.03, 0.05)	0.545	0.01 (-0.03, 0.05)	0.771	0.02 (-0.03, 0.07)	0.475
**Arts/crafts**						
Low	0.02 (-0.02, 0.06)	0.233	0.01 (-0.03, 0.04)	0.682	0.00 (-0.05, 0.05)	0.991
High	0.00 (-0.04, 0.05)	0.844	-0.02 (-0.06, 0.03)	0.449	**0.06 (0.01, 0.12)**	**0.023**
**Digital arts**						
Low	0.01 (-0.04, 0.05)	0.782	-0.01 (-0.05, 0.03)	0.610	-0.03 (-0.09, 0.02)	0.237
High	0.04 (-0.03, 0.10)	0.248	0.04 (-0.01, 0.09)	0.157	-0.05 (-0.11, 0.02)	0.196
**Gardening**						
Low	**-0.07 (-0.11, -0.02)**	**0.003**	**-0.05 (-0.09, -0.01)**	**0.014**	0.04 (-0.01, 0.09)	0.102
High	**-0.05 (-0.10, -0.01)**	**0.028**	-0.01 (-0.06, 0.05)	0.802	**0.06 (0.00, 0.12)**	**0.033**
**Watching TV**						
Low	0.04 (-0.01, 0.09)	0.119	-0.02 (-0.07, 0.03)	0.461	-0.01 (-0.07, 0.06)	0.868
High	**0.05 (0.01, 0.10)**	**0.013**	-0.02 (-0.06, 0.02)	0.405	0.00 (-0.05, 0.04)	0.852
**Listening to music**						
Low	-0.01 (-0.04, 0.03)	0.709	-0.01 (-0.05, 0.02)	0.355	0.00 (-0.04, 0.04)	0.930
High	-0.02 (-0.05, 0.02)	0.410	-0.01 (-0.05, 0.02)	0.440	0.00 (-0.04, 0.04)	0.930
**Woodwork/DIY**						
Low	0.02 (-0.04, 0.09)	0.489	0.02 (-0.05, 0.08)	0.590	-0.05 (-0.11, 0.02)	0.167
High	0.00 (-0.05, 0.05)	0.932	-0.02 (-0.07, 0.02)	0.353	**0.10 (0.03, 0.18)**	**0.004**
**Other hobbies**						
Low	0.01 (-0.03, 0.06)	0.563	0.02 (-0.02, 0.07)	0.322	-0.03 (-0.09, 0.02)	0.264
High	0.01 (-0.03, 0.06)	0.552	0.00 (-0.04, 0.05)	0.878	-0.02 (-0.08, 0.04)	0.496

Note. Time spent doing activities measured on the last weekday. Low = less than 30 mins doing activity during the day. High = 30 mins or more spent on activity during the day. Both low and high were compared to doing none of this activity. Models show associations between changes in time spent on leisure activities and changes in mental health and wellbeing across the follow-up period (6th April – 6th September 2020). Outcomes were standardised, so coefficients represent changes in standard deviation units.

Table S5. Interaction terms from fixed effects models testing whether the associations between time spent on leisure activities and outcomes differed according to baseline employment status.

	**Depressive symptoms**		**Anxiety symptoms**		**Life satisfaction**	
	Coef (95% CI)	p	Coef (95% CI)	p	Coef (95% CI)	p
**Reading for pleasure**	0.01 (-0.22, 0.25)	0.904	-0.07 (-0.27, 0.14)	0.528	-0.03 (-0.15, 0.09)	0.646
**Arts/crafts**	-0.14 (-0.39, 0.11)	0.263	0.01 (-0.22, 0.23)	0.935	0.04 (-0.08, 0.16)	0.480
**Digital arts**	0.27 (-0.06, 0.60)	0.107	0.17 (-0.07, 0.42)	0.161	0.04 (-0.11, 0.19)	0.590
**Gardening**	-0.11 (-0.37, 0.16)	0.437	-0.31 (-0.57, -0.04)	0.024	0.03 (-0.10, 0.16)	0.634
**Watching TV**	-0.10 (-0.34, 0.15)	0.445	-0.02 (-0.23, 0.19)	0.882	0.03 (-0.08, 0.14)	0.571
**Listening to music**	0.13 (-0.08, 0.35)	0.222	0.08 (-0.10, 0.25)	0.380	0.01 (-0.08, 0.11)	0.776
**Woodwork/DIY**	0.05 (-0.26, 0.36)	0.752	-0.09 (-0.34, 0.16)	0.488	0.02 (-0.13, 0.18)	0.766
**Other hobbies**	-0.10 (-0.36, 0.15)	0.424	-0.14 (-0.37, 0.09)	0.234	0.06 (-0.06, 0.19)	0.323

Note. Leisure activities were treated as continuous to test whether there was overall evidence for an interaction with baseline employment status. Outcomes were standardised, so coefficients represent changes in standard deviation units.

Table S6. Fixed effects models including a combined index of time spent on creative hobbies (arts/crafts, digital arts, woodwork/DIY).

	**Depressive symptoms**		**Anxiety symptoms**		**Life satisfaction**	
	Coef (95% CI)	p	Coef (95% CI)	p	Coef (95% CI)	p
Creative hobbies						
Low	0.03 (-0.01, 0.07)	0.093	0.01 (-0.03, 0.05)	0.576	-0.02 (-0.07, 0.02)	0.305
High	0.02 (-0.02, 0.06)	0.254	0.00 (-0.04, 0.03)	0.796	0.03 (-0.01, 0.07)	0.130
Reading for pleasure						
Low	0.01 (-0.03, 0.05)	0.526	0.02 (-0.02, 0.06)	0.272	0.01 (-0.03, 0.06)	0.488
High	0.02 (-0.02, 0.06)	0.293	0.01 (-0.03, 0.05)	0.598	0.01 (-0.03, 0.06)	0.555
Gardening						
Low	**-0.07 (-0.11, -0.02)**	**0.003**	**-0.05 (-0.09, -0.01)**	**0.015**	0.04 (-0.01, 0.09)	0.108
High	**-0.05 (-0.09, 0.00)**	**0.042**	0.00 (-0.06, 0.05)	0.863	**0.07 (0.01, 0.12)**	**0.028**
Watching TV						
Low	0.04 (-0.01, 0.09)	0.150	-0.02 (-0.07, 0.03)	0.460	0.00 (-0.07, 0.06)	0.950
High	**0.06 (0.01, 0.10)**	**0.010**	-0.02 (-0.06, 0.03)	0.486	-0.01 (-0.06, 0.04)	0.814
Listening to music						
Low	-0.01 (-0.04, 0.03)	0.771	-0.01 (-0.04, 0.02)	0.432	0.00 (-0.05, 0.04)	0.841
High	-0.02 (-0.05, 0.02)	0.396	-0.01 (-0.05, 0.02)	0.513	0.00 (-0.04, 0.04)	0.915
Other hobbies						
Low	0.02 (-0.02, 0.07)	0.330	0.03 (-0.02, 0.07)	0.205	-0.05 (-0.10, 0.01)	0.108
High	0.02 (-0.03, 0.06)	0.525	0.01 (-0.04, 0.05)	0.818	-0.02 (-0.09, 0.04)	0.446

Note. Time spent doing activities measured on the last weekday. Low = less than 30 mins doing activity during the day. High = 30 mins or more spent on activity during the day. Both low and high were compared to doing none of this activity. Models show associations between changes in time spent on leisure activities and changes in mental health and wellbeing across the follow-up period (6th April – 6th September 2020). Outcomes were standardised, so coefficients represent changes in standard deviation units.

Bone JK, *et al. J Epidemiol Community Health* 2023; 77:293–297. doi: 10.1136/jech-2022-219653

References

Allison, P. D. (2009). *Fixed effects regression models*. SAGE Publications.

Bu, F., Steptoe, A., Mak, H. W., & Fancourt, D. (2021). Time use and mental health in UK adults during an 11-week COVID-19 lockdown: a panel analysis. *The British Journal of Psychiatry*, 1–6. https://doi.org/10.1192/bjp.2021.44

Chowdhury, S., Khare, M., & Wolter, K. (2007). Weight trimming in the national immunization survey. In *Proceedings of the Joint Statistical Meetings, Section on Survey Research Methods* (pp. 2651–2658). American Statistical Association.

Hainmueller, J., & Xu, Y. (2013). Ebalance: A stata package for entropy balancing. *Journal of Statistical Software*, *54*(7), 1–18. https://doi.org/10.18637/jss.v054.i07

Kroenke, K., Spitzer, R. L., & Williams, J. B. W. (2001). The PHQ-9: validity of a brief depression severity measure. *J Gen Intern Med*, *16*(9), 606–613.

Potter, F., & Zheng, Y. (2015). Methods and Issues in Trimming Extreme Weights in Sample Surveys. In *Proceedings of the American Statistical Association, Section on Survey Research Methods* (pp. 2707–2719). American Statistical Association.

Spitzer, R. L., Kroenke, K., Williams, J. B. W., & Löwe, B. (2006). A brief measure for assessing generalized anxiety disorder: The GAD-7. *Archives of Internal Medicine*, *166*(10), 1092–1097. https://doi.org/10.1001/archinte.166.10.1092

StataCorp. (2019). *Stata Statistical Software: Release 16*. StataCorp LP.

US Census Bureau. (2021). *US Population*. https://www.census.gov/topics/population.html
